# Updates on Biomaterials Used in Total Hip Arthroplasty (THA)

**DOI:** 10.3390/polym15153278

**Published:** 2023-08-02

**Authors:** Liliana Savin, Tudor Pinteala, Dana Nicoleta Mihai, Dan Mihailescu, Smaranda Stefana Miu, Mihnea Theodor Sirbu, Bogdan Veliceasa, Dragos Cristian Popescu, Paul Dan Sirbu, Norin Forna

**Affiliations:** 1 Department of Orthopedics and Traumatology, Faculty of Medicine, “Grigore T. Popa” University of Medicine and Pharmacy, 700115 Iasi, Romania; liliana.savin@umfiasi.ro (L.S.); dan.mihailescu1@umfiasi.ro (D.M.); sirbu_theo@yahoo.com (M.T.S.); velbogdan@yahoo.com (B.V.); dragospopescu1975@yahoo.com (D.C.P.); pdsirbu@yahoo.com (P.D.S.); norin.forna@umfiasi.ro (N.F.); 2 Department of Orthopedics, Clinical Rehabilitation Hospital, 700661 Iasi, Romania; dana-nicoleta.stratulat-mihai@umfiasi.ro; 3 Department of Protheses Technology, Faculty of Dental Medicine, “Grigore T. Popa” University of Medicine and Pharmacy, 700115 Iasi, Romania; 4 Department of Rehabilitation, Clinical Rehabilitation Hospital, 700661 Iasi, Romania; smaranda.miu@gmail.com

**Keywords:** total hip arthroplasty, metal, ceramic, polyethylene, biomaterials

## Abstract

One of the most popular and effective orthopedic surgical interventions for treating a variety of hip diseases is total hip arthroplasty. Despite being a radical procedure that involves replacing bone and cartilaginous surfaces with biomaterials, it produces excellent outcomes that significantly increase the patient’s quality of life. Patient factors and surgical technique, as well as biomaterials, play a role in prosthetic survival, with aseptic loosening (one of the most common causes of total hip arthroplasty failure) being linked to the quality of biomaterials utilized. Over the years, various biomaterials have been developed to limit the amount of wear particles generated over time by friction between the prosthetic head (metal alloys or ceramic) and the insert fixed in the acetabular component (polyethylene or ceramic). An ideal biomaterial must be biocompatible, have a low coefficient of friction, be corrosion resistant, and have great mechanical power. Comprehensive knowledge regarding what causes hip arthroplasty failure, as well as improvements in biomaterial quality and surgical technique, will influence the survivability of the prosthetic implant. The purpose of this article was to assess the benefits and drawbacks of various biomaterial and friction couples used in total hip arthroplasties by reviewing the scientific literature published over the last 10 years.

## 1. Introduction

Total hip arthroplasty (THA) significantly changed the treatment of patients handicapped by arthritis in the 1960s, producing favorable long-term results and thus becoming one of the most successful orthopedic surgical interventions [1] and one of the top five most common and fastest-growing procedures [2].

Nowadays, it is estimated that, annually, more than one million patients require an artificial hip to replace the affected joint [3] and, in the upcoming years, the volume and demand for total hip arthroplasty will increase due to increased demand for improved mobility and quality of life in an aging population [4] as well as younger patients who want to improve their quality of life, which typically includes physically demanding activities [5,6]. In addition, it is anticipated that, by 2030, approximately 50% of all arthroplasties would be performed on patients under 65 years, with a ratio of 1.5–2.0/1 female-to-male, except in South Korea where a 7–8/1 female-to-male ratio is estimated [7]. Moreover, THA has significantly increased in recent years, with an expected 173% rise from 2005 to 2030, translating to 572,000 hip arthroplasties performed annually by 2030 [8,9]. Furthermore, according to a recent study involving the Organization for Economic Co-operation and Development (OECD), over the following 35 years, hip arthroplasties will continue to increase dramatically, which is reflected in rising numbers of hip implants—from 1.8 in 2015 to 2.8 million in 2050, with an annual growth rate of 1.2% and an average hip implant usage rate incidence from 184 to 275 per 100,000 people. According to the same study, in Australia, Ireland, Norway, Switzerland, and other countries, the usage of hip implants is anticipated to increase dramatically between 2015 and 2050, by a range of +95% to +120% [10]. Despite being a successful procedure, THA puts a lot of strain on individuals and national healthcare systems; as a result, the importance of health technology assessment for medical devices will grow in terms of dependability and quality control [2]. In this context, bioengineering advances have contributed to the development of hip prostheses, better materials, and designs that give a greater range of motion while also improving stability and reducing friction [1]. Along with the improvement of biomaterials used in THA, computer-assisted surgery is expected to provide contributions in comparison to classical THA surgery, having advantages such as precise implant placement and decreasing complication rates, with minimally invasive surgery reducing soft-tissue injury and allowing for faster discharge and recuperation [11,12]. 

**Figure 1:**
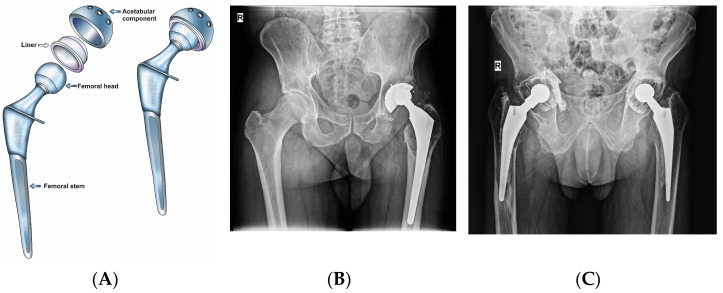
(**A**) Main components of a total hip prosthesis, (**B**) male patient 15 years post left non-cemented total hip arthroplasty (MoP) showing polyethylene wear and particle disease around the left acetabular and trochanteric regions, (**C**) male patient 19 years post bilateral cemented total hip arthroplasty showing bilateral aseptic loosening (images from the archive of the Orthopedics and Traumatology Department, Clinical Rehabilitation Hospital of Iași, Romania).

The prosthetic hip implant includes two metal components fixed in the bone: the stem inserted into the femoral canal after its preparation with specific rasps and the acetabular shell fixed in the pelvis after reaming with consecutive acetabular reamers (Figure 1).

The prosthetic head attached to the femoral stem articulates with the liner inside the metal acetabular implant, thus causing movement of the hip joint. The prosthetic articular surfaces of the hip can be made of different materials: metal or ceramic head; ceramic, metal, or polyethylene acetabular insert. The mobility between these articular surfaces creates a frictional element that causes wear of the acetabular insert over time and the release of particles in the joint [11,13,14]. Wear particles inside the hip joint cause the appearance of an inflammatory reaction, called particle disease, and peri-implant osteolysis and aseptic loosening of the prosthesis, which requires revision surgery [15]. 

The incidence of total hip arthroplasty revision is estimated to increase from 43 to 70% in the 2014–2030 period [9,16]. Some studies show that the survival of the prosthetic implant decreases significantly after 15–25 years [17], and aseptic loosening is one of the most frequent causes of revision [9,18]. A recent systematic review by Kennedy et al. [19] highlighted the fact that 23% of revision arthroplasties were caused by aseptic loosening.

With the increase in the patient’s quality of life, we are witnessing an increase in the indication for surgery, with age no longer being a contraindication or a limitation of the intervention; thus, the indication for THA is addressed to an increasingly younger age, this type of patient having the risk of multiple revisions throughout life. The need for the survival of the prosthetic implant for as long as possible has led to changes in surgical technique and multiple biomechanical studies, as well as the discovery and development of new biomaterials and implants [11,20].

In 1987, Donaruma et al. [21] defined biomaterial as “a synthetic or natural biocompatible material that comprises whole or part of a living structure or a biomedical device which performs, augments or replaces a function that has been lost through disease or injury with no negative effects on the biological environment”. An ideal biomaterial must be biocompatible, have a low coefficient of friction, be resistant to corrosion, and have mechanical strength [11,20]. Increasing the longevity and stability of the implant can be helped by reducing the release of polyethylene particles at the joint level, and by improving the quality of the biomaterials used in the mobile joint components. The current study aimed to review the advantages and disadvantages of biomaterials used as bearing surfaces and the differences between different friction couples used in total hip arthroplasty.

## 2. Background

The first data on degenerative changes of the hip appeared in archaeological and paleontological research [22,23,24]. In the medieval period, the only treatment for hip osteoarthritis was orthopaedic (non-surgical) [25]. Surgical treatment for hip arthritis was described for the first time by Henry Park et al. in 1782 [26] only as an excisional arthroplasty; the first surgical intervention excised the entire femoral head with the aim of the subsequent appearance of a “bone callus” [26]. In 1826, John Rhea Barton [27] performed the first osteotomy of a hip in ankylosis with poor results, and only in 1885 did the idea of interpositional arthroplasty (with adipose tissue) appear for the first time [28]. The first interpositional arthroplasty with synthetic material was described by Marius Smith-Petersen in 1923 [29], when he placed a glass mould between the femoral head and the acetabulum. Later, different interpositional materials were used, such as Vitallium, Bakelite, and Pyrex [22,24].

The first arthroplasty that replaced the articular surface was performed by Pierre Delbet in 1919 [22,24], using a rubber prosthesis instead of the femoral head. In 1938, the first total hip arthroplasty was described, in which stainless steel components that were fixed to the bone with screws and bolts were used, but the results were unsatisfactory [30]. In 1940, Austin Moore [31] created a Vitallium stem inserted into the femoral canal, still used today for elderly patients with femoral neck fractures, which replaces only one part of the joint.

The father of modern hip arthroplasty was Sir John Charnley, who introduced the concept of low friction arthroplasty—metal on polyethylene (MoP) [32]. The concept was based on three distinct ideas [33]: low friction couple arthroplasty, components fixed with acrylic cement to the bone, and the use of high-density polyethylene as a bearing material. The size of the metal femoral head was reduced from 40 mm to 22.225 mm, obtaining a limited mobility of 90° [22]. Muller increased the size of the head to 32 mm, achieving better mobility (about 106°) but with complications of aseptic loosening and osteolysis [22]. Thus, Charnley understood the need to use another bearing material, which was ultrahigh molecular weight polyethylene (UHMWPE) [22].

Different authors have started using different combinations of materials for both cemented and non-cemented components: Peter Ring used an uncemented prosthesis with a metal-on-metal friction couple (MoM) [34], and the alumina ceramic-on-ceramic (CoC) bearing was introduced by Boutin [35]. These methods to reduce polyethylene wear consisted in improving the properties of the materials and the design of bearing surfaces. The increase in the cross-linking degree of UHMWPE determined the increase in strength and rigidity with a decrease in wear [36] but, at the same time, increased oxidation leads to a decrease in fracture and fatigue resistance with the potential for increased wear [37]. The introduction of antioxidants, such as vitamin E, favoured the decrease in wear and the increase in oxidation resistance [38,39,40]. Over time, multiple changes have appeared in the prosthetic femoral head. Changing the classic CoCrMo with ceramic materials, or coating with different alloys like diamond-like carbon (DLC) [41], tantalum [42], or titanium nitride [43], has led to an increase in hardness and a decrease in the roughness of the articular surface of the femoral head, thus reducing the decrease in polyethylene wear [44]. With these improvements, the antimicrobial characteristics and the capacity to sustain cell adhesion and proliferation have provided prostheses with good osseointegration [41].

## 3. Bearing Surfaces and Biomaterials Used in Primary Total Hip Arthroplasties

Hip arthroplasties vary in terms of component design and biomaterials used [45]. An important characteristic of the prosthesis is the bearing surface and, in modern times, it can be made of metal-on-metal (MoM), metal-on-polyethylene (MoP), ceramic-on-ceramic (CoC), or ceramic-on-polyethylene (CoP) materials, where the polyethylene can be ultra-high molecular weight polyethylene (UHMWPE) or highly cross-linked polyethylene [HXLPE] [45,46]. The most often utilized bearing surfaces for THA are highly cross-linked polyethylene (HXLPE), ceramic, ultra-high molecular weight polyethylene (UHMWPE), and metal. However, there is no consensus on the best femoral head material, but the longest clinical monitoring of any combination is metal-on-HXLPE; more recently considered a superior option, oxidized zirconia or alumina ceramic femoral heads have been discussed as a better choice [47].

### 3.1. Polymers

Polymers are used in hip arthroplasty in the liner, inside the metal acetabular cup. The use of different types of polymers has an impact on the wear, over time, of the mobile implant and subsequently on the stability of the prosthetic implant (Table 1).

Polymethyl methacrylate (PMMA) was introduced by Charnley in the 1960s [48,49,50] as a bone cement to fix components for THA into the living bone with good long-term outcomes [51]; however, later studies have shown that implant loosening and displacement have been linked to PMMA cement due to the exothermic temperature of the polymerization process of methyl methacrylate monomer in the presence of an initiator, resulting in the release of a toxic monomer, thus causing bone necrosis and therefore implant loosening [52].

Polytetrafluoroethylene (Teflon) (PTFE) is a hydrophobic and thermally stable fluoropolymer of tetrafluoroethylene that has a very high wear coefficient; this was used for the first time by Charnley et al. [32] as an alternative to polymethyl methacrylate [53]. Affatato et al. [20] described a wear rate of 0.5 mm/month and the production of a voluminous mass of loose particles [54]. Due to its low coefficient of friction, PTFE was considered to be comparable to the hyaline cartilage seen in natural joints but was restricted by early catastrophic wear that created considerable local tissue responses that aided in osteolysis, aseptic loosening, and clinical failure [55].

Ultra-high molecular weight polyethylene (UHMWPE) is an engineering polymer that was launched in THA in the middle of the 1960s as a replacement for the unsuccessful PTFE [56]. It has become the most often used bearing material with ceramic or metallic counter surfaces dedicated to THA [32,54,57,58,59]. The molecular weight of UHMWPE is at least 1 million g/mole, while the degree of polymerization is 36,000 with a crystallinity of 50–55%, according to the International Standard Organization (ISO 11542). The American Society for Testing and Materials stated that its molecular weight is over 3.1 million g/mole, while the degree of polymerization is 110,000 and its properties include high wear-resistance, toughness, durability, and biocompatibility [17,59,60,61].

It is concerning how often THA fails over time because of wear problems that shorten UHMWPE’s longevity, and the release of polyethylene particles may result in peri-implant osteolysis and aseptic loosening. UHMWPE wear is one of the most serious post-surgical concerns and the development of UHMWPE is essential for prolonging survival time after THA [17,18,56,59,61,62,63,64,65].

Today, the primary problem of the oxidation of UHMWPE has been significantly decreased by replacing the air with gas or low oxygen conditions during radiation. At the same time, in sterilizing methods, wear is controlled by cross-linking [59,61]. Also, in a recent study, Slouf et al. [56] predicted that oxidative degradation will increase once the median lifetime of the UHMWPE liner in vivo increases and a study of Ishida’s group also predicted this [66].

Highly cross-linked polyethylene [HXLPE] was developed in the 1990s to decrease the wear of UHMWPE when manufacturers such as Stryker, Zimmer Biomet, Kyocera, DePuy Synthes, and Smith & Nephew placed on the market first generation trade-marks like Crossfire, ArCom XL, Aeonian, Marathon and XLPE, respectively, which were produced by 50–100 kGy radiation and stabilization with remelting or annealing. Later, the HXLPE second generation was produced by using sequential radiation and annealing [67,68]. It is advised to utilize remelted HXLPE liners with a minimum thickness of 7 mm for weight bearing and the minimal thickness permitted at the rim is recommended to be at least 4.8 mm [69]. In a more recent study, Fransen et al. [70] showed that the mid-term results of using HXLPE liners in association with 36 mm heads are safe. According to property studies, as the irradiation dose increases, the mechanical properties and material’s plasticity decrease with the appearance of free radicals and implant fragility; in addition, increasing cross-link density leads to a reduction in propagation resistance [54,59,71].

Later, the combination of cross-linking and heat treatment was proposed, which led to a decrease in free radicals and an increase in resistance to oxidation and wear [20,72,73,74]. Free radicals react with oxygen, causing the splitting of polymer chains and resulting in the release of carboxylic acids and ketones, which reduce the performance of the polyethylene [67,69,75,76]. To increase low friction properties by increasing the wettability, the surface of prostheses was grafted with phosphorylcholine derivatives [77]. 

It should be mentioned that Devane and colleagues [78,79] demonstrated that HXLPE has less wear when compared to conventional UHMWPE 10 years following primary THA, and Kim [80] highlighted a penetration of 0.022 mm/year when using a delta ceramic head. Despite all this information, there is still no information in the registry data referring to long-term outcomes with second-generation HXLPE [59]. 

Until now, the most-used bearing coupling is the cobalt-chromium femoral head that articulates with an HXLPE liner, since it shows extended durability and reduced wear rates with good safety in various longitudinal inspections [45,81].

Vitamin E-blended UHMWPE or HXLPE arose from the need to decrease the production of free radicals. Vitamin E is a biocompatible antioxidant, with multiple beneficial roles (anti-inflammatory, antibacterial), that protects the polymer from oxidative degradation [82,83,84]. A-tocopherol is inserted into the material before or after cross-linking irradiation and before sterilization (bulk diffusion technique) [85,86,87]. Numerous trials have shown mid-term effects after THA with HXLPE in combination with vitamin E, although the findings were inconsistent. For example, in a 2019 study, Galea [88] compared the wear occurring 5 years after THA in patients with a UHMWPE insert compared to those with a vitamin E-blended polymers insert, highlighting a greater penetration of the prosthetic head into the insert in the first patients, while studies of Kjærgaard [89] and Hasegawa [59] found no changes in hips with and without dispersed vitamin E after 5 years. In a very recent review, Spece et al. [84] showed that, in every study that examined polyethylene (UHMWPE or HXLPE) with and without vitamin E, HXLPE blended with vitamin E was shown to be clinically successful for THA applications, demonstrating good clinical results and reduced or equal wear rates compared to standard UHMWPE and HXLPE without vitamin E, with hip implant wear rates of less than 0.1 mm/year and, in the majority of cases, of less than 0.05 mm/year. In addition, no revisions for vitamin E-blended HXLPE components for osteolysis or poor outcomes related to vitamin E integration were reported.

Polyether-ether-ketone [PEEK] is a biocompatible polymer that promises a much lower release of wear particles when used as a bearing surface [90,91] but with few clinical studies to date. PEEK has an elastic modulus closer to that of bone, thereby decreasing the stress shielding effect seen in metal [90]. The advantages of PEEK are as follows: high thermal stability, toughness, rigidity, creep resistance, ease of processing, self-lubrication, and high abrasion resistance [92].

Poly 2-methacryloyloxyethyl phosphorylcholine [PMPC] is formed by grafting poly (2-methacryloyloxyethyl phosphorylcholine) (MPC) onto an HXLPE using photoinduced polymerization [93]. The articular surface of HXLPE is covered by a coating that simulates articular cartilage, having a massive effect on the reduction of wear particles [54,93].

Polycarbonate-urethane [PCU] is a hydrophilic implant with an elasticity close to that of human cartilage. The advantages are as follows: biostability, high resistance to hydrolysis, oxidation, and calcification, absent biodegradation, low wear rate, and high corrosion resistance [28,54,94].

### 3.2. Metals

Metals are used in THA both for the fixed components (femoral stem and acetabular component) as well as for the mobile components (femoral head and liner). It should be mentioned that MoM THA is no longer utilized [45,46,95], because metal wear debris may increase metal ion levels in serum and can generate hypersensitivity responses in the soft tissues, with harmful systemic consequences [96] such as pseudotumor formation and aseptic lymphocyte-dominated vasculitis-associated lesions (ALVAL), leading to eventual soft tissue and bone destruction [97]; due to this, MoM implants have been replaced more and more with metal-with-polymer (MoP) THA implants [11].

Stainless steel is a carbon-based iron alloy that contains Cr, Ni, Mo, Mn and C [54]. It is a metal with high oxidation resistance but is rarely used in hip arthroplasty due to abrasive wear [98].

Cobalt chromium molybdenum alloys (CoCrMo) are composed of 60–70% Co, 25–30% Cr, and 5–7% Mo [54] and are frequently used in the prosthetic head component. The greatest risk is posed by the cytotoxicity and carcinogenesis given by the particles released in the joint in patients with MoM friction torque [83].

Titanium alloys (Ti-6Al-4 V) are used especially in femoral stems and acetabular cups due to their biocompatibility and mechanical properties [54]. Their disadvantage is due to their low resistance to wear [98], which is why they are not used for the prosthetic head.

Zirconium alloy (Zr-2.5Nb) is a new alloy used in the production of the prosthetic head. Oxidized zirconium implants with lower wear can improve the longevity of total hip implants [99,100]. Oxidized zirconium is a metal alloy of zirconium with a ceramic surface, representing the transformation of the metal underlayer due to the diffusion of oxygen into zirconium when heated, thus producing a ceramic layer of zirconium oxide [101,102,103,104]. Higher values of hardness and lower values of surface roughness are reported for ceramics, while metals have high values of fracture toughness and fatigue strength [105].

In order to decrease the release of metal ions during the friction of the joint surfaces, different metal alloy surface coatings were tested. All these covers, no matter if they are Titanium nitride (TiN) [106], Silicon nitride (Si3N4) [107], Diamond-like carbon (DLC) [108], Aluminium coating [109], Nanocrystalline diamond (NCD) [110], or polycrystalline diamond, have a low coefficient of friction, high wear resistance, scratch resistance, and higher fracture toughness. Because of its mechanical characteristics and biocompatibility, polycrystalline diamond, compared to metal and ceramic hard-on-hard bearings, has the potential to become the preferred material for hard-on-hard bearings [111]. Recently, it was demonstrated that magnesium alloys, AZ31, possess properties similar to human bone and, in combination with silicon nitride (Si3N4), present improved biomechanical characteristics, being a superior choice for CoCrMo, CoCr, and titanium alloys [112]. Also, tantalum has been shown in recent investigations to have strong biocompatibility and osseointegration characteristics and may be utilized to not only replace significant bone abnormalities but also to offer initial and long-term stability for prostheses [113]. Overall, the combined mechanical qualities of metal and ceramics, according to a few studies, boost the prosthetic head’s resistance to fracture and decrease the release of metal ions when the acetabular insert rubs against it. In this context, an improved choice is to spray hydroxyapatite on the prosthetic head, assuring bone ingrowth [114,115]. The characteristics of the most-used metal heads are presented in Table 2.

### 3.3. Ceramic Materials

Ceramic materials were introduced in THA approximately 50 years ago [127] with the aim of reducing the wear of the acetabular insert and peri-implant osteolysis [127,128]. Ceramic materials can be used for making mobile components, the prosthetic head, and the acetabular liner, thus resulting in two ceramic-on-ceramic or ceramic-on-polyethylene friction couples, each with its advantages and disadvantages. Component fracture risk was a worry in CoC arthroplasties; however, with the newer generations of implants, when the complication rates were reduced, the use of CoC has not considerably decreased [45,95] and remains one of the most effective ways to prevent liner wear with wear rates of less than 0.001 mm/year compared to 0.072 mm/year in ceramic-on-UHMWPE (CoUHMWPE), 0.30 mm/year in ceramic-on-HXLPE (CoHXLPE), and 0.042 mm/year in metal-on-HXLPE (MoHXLPE) [129]. Furthermore, a very recent study from 2023 found the Delta ceramic bearing to be a viable alternative for primary and revision THA, with favourable mid-term outcomes, satisfactory survival, and a low complication rate [130].

Alumina is a ceramic with high biocompatibility. The commercial product is Biolox, with a high elastic modulus (400 GPa) overpassing that of the cortical bone (300 GPa); also, it has low deformation values [131]. The advantages of this ceramic are given by high hardness values, a low friction coefficient, and high wear resistance; the wear rate is 4000 times lower when compared to MoP [131]. The weak point is its mechanical fragility and the possibility of breakage and, if used in a CoC couple, it produces a noise [132]. Alumina ceramic-on-ceramic has shown at least 20 years of implant survival and minimal complications after THA [133]. 

Zirconia has the same advantages as alumina but with good resistance to breaking [54,134]. 

Zirconia-toughened alumina (ZTA) is a combination of two materials: Zirconium is incorporated into the aluminium matrix to increase hardness [128]. This product, also called Biolox delta, combines the strength and hardness of zirconia with the wear resistance of alumina [132] but with the same weak spot represented by squeaking [135]. A reduction to 0.003% of the fracture incidence of the ZTA implant was reported, a significantly lower value when compared to 0.021% for the alumina one [136].

Oxidized zirconium head-on crosslinked polyethylene is a modern bearing coupling but more studies are needed due to in vivo lower wear parameters [137].

Sapphire is a mineral that contains 99.99% aluminium oxide and traces of chromium, titanium, iron, vanadium, and magnesium [54]. A few studies show good resistance to wear and a low coefficient of friction [54,138]. The characteristics of the most commonly used ceramics in THA are presented in Table 3.

Silicon nitride, silicon carbide and diamond-like carbon as non-oxide ceramics are considered to be the new generation of materials used in hip prosthetics, particularly in the manufacture of acetabular cups, due to their excellent biocompatibility, osteointegration, and tribological and mechanical properties, but all three materials need more study. However, silicon nitride is the nearest to commercialization, through businesses such as Amedica Corp. and SyntX Technologies [14].

## 4. The Choices of the Bearing Surfaces

**Figure 2:**
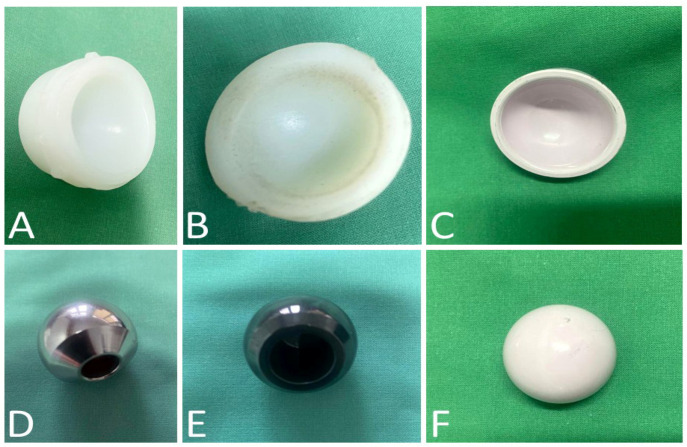
(**A**) HXLPE liner, (**B**) wear areas spotted on UHMWPE liner, (**C**) ceramic liner, (**D**) CoCrMo metallic head, (**E**) Oxinium head, (**F**) ceramic head (Orthopedics and Traumatology Department, Clinical Rehabilitation Hospital of Iași, Romania).

The use of bearing surfaces in total hip arthroplasty for young and active patients remains controversial. Despite the popularity of polyethylene among surgeons due to its ease of use, the risk of wear and aseptic loosening has led to increased interest in researching the best bearing surfaces. As an alternative to conventional metal-on-polyethylene (MoP), metal-on-metal (MoM), ceramic-on-high cross-linked polyethylene (CoHXLPE), or ceramic-on-ceramic (CoC) have become possibilities when choosing the most suitable ported surfaces (Figure 2).

### 4.1. Metal-on-Polyethylene (MoP)

MoP is the most-used bearing couple combination in hip arthroplasty, developed by Sir John Charnley [32]. In this type of arthroplasty, a metal head is used that articulates with a simple polyethylene insert/cross-linked/with vitamin E (Figure 2D). Polyethylene is frequently used in orthopaedics due to the advantages of low cost, good wear rate, and good impact receiver (shock absorber quality), being a good choice for the elderly patient [139].

Multiple studies have shown a decrease in the release of polyethylene particles in the cases of XLPE and HXLPE compared to the case of UHMWPE [78] (Figure 2A,B). Kuzyk et al., in a systematic review, highlighted a much greater penetration of the prosthetic head in UHMWPE [0.137 mm/year] compared to XLPE [0.042 mm/year] [140]. Wear rates for UHMWPE of 0.1–0.2 mm/year were reported with large particles, compared to the wear rate for HXLPE of 0.051–0.25 mm/year with small particles [140].

Vitamin E-supplemented cross-linked polyethylene (VE-HXLPE) has been implemented recently in total hip arthroplasty due to its improved tribological properties by the decrease of liners oxidation [82,141]. 

Various research groups investigated this approach and assessed the possibility of the improvement of the wear behaviour of the vitamin-E supplemented liners of a hip prosthesis in total hip arthroplasty. While Takahashi et al. [142] assessed the wear rates of both categories of liners (vitamin-E blended and infused highly cross-linked polyethylene) using an exponential model and found the absence of any significant difference, the results vary throughout the literature. However, some reports show higher values of the restoring forces against a uniaxial strain of cross-linked polyethylene, as well as higher values of the degree of surface orientational randomness; these data explain the decrease of wear in in vivo studies due to strain softening [142]. Uetsuki et al. [143] highlighted the decrease of the wear rates in biomechanical tests of VE-HXLPE. Three research groups demonstrated that VE-HXLPE has significantly improved mechanical properties compared to conventional UHMWPE [144,145,146]. A test group followed for 1, 2, and 3 years postoperative showed that E-blended liners have less cumulative penetration of the femoral head when compared to a conventional cup, with 0.200 mm cumulative penetration at 3 years for an HXLPE/VitE cup versus 0.317 mm for a conventional cup [145]. Elbardesy et al. [147] found the absence of significant differences at 1-year follow-up but significantly less femoral hip penetration at 2- and 5-years follow-up for patients with vitamin E-blended liners when compared with conventional liners. These studies concluded that vitamin E-supplemented liners have the advantages of preventing osteolysis and implant loosening, and are a necessity for revision surgery. However, the authors highlight the need for long-term follow-up data. These long-term studies are requested also by the research groups that did not find any significant differences between conventional and E-blended HXLPE liners. A prospective study, conducted on 324 patients, evaluated cup wear behaviour using the RayMatch^®^ analysis software. At 5-year follow-up, wear rates of non-vitamin E-supplemented liners and vitamin-E supplemented liners were comparable [148]. Also, Nielson [149] used femoral head penetration measured on radiographs to analyse the true abrasive wear of the polyethylene liner [VE-HXLPE vs. conventional] and did not find significant differences between these categories of liners.

Oxidized zirconium (OxZr/OxZi) or Oxinium™ [Smith & Nephew, Memphis, TN, USA] is a new metal alloy that is transformed to ceramic using a complex process; it is considered an alternative bearing surface and is used for femoral heads in total hip arthroplasty (Figure 2E). Recent research shows that there is a lower [150,151] or similar [105,152,153] total wear and wear rate of the polyethylene insert when comparing CoCr and OxZr femoral heads in total hip arthroplasty. On the other hand, it seems that, when comparing OxZr and ceramic femoral heads with either crosslinked polyethylene [HXLPE] or conventional polyethylene, the wear rate of the polyethylene is smaller with the OxZr heads [137]. Also, the wear of the HXLPE is similar when using either metal or ceramic femoral heads [154] and, furthermore, it seems that there is no difference in steady wear rate between the ceramic, OxZr, and CoCr when using HXLPE [155].

### 4.2. Metal-on-Metal (MoM)

The metal–metal couple (MoM) used as metal heads in metal inserts focused the interest of research groups and orthopaedic surgeons. Literature data report lower osteolysis and wear rates due to the decreased diameter of particles and lower values of friction [156]. In 2006–2008, 10% of all arthroplasties in the UK were performed by using the MoM prosthesis [157], with MoM studies showing satisfactory short- and medium-term survival [158,159].

However, a few research groups highlighted significant concerns regarding biocompatibility. Adverse reactions to the metal particles released by wear processes were noted due to the destruction of the periprosthetic soft tissues by lymphocytes [160]; these led to pain, a loosening process, and an increased rate of revisions. Second, wear particles in the form of cobalt and chromium ions were detected throughout the body [161]. Also, granulomas were found in the liver and spleen [162]. Increased chromosomal translocation was reported in lymphocytes [163]. Despite there being no evidence of this neoplasia occurrence [164], the British Orthopaedic Association [BOA] recommended the avoidance of hip replacement with large head diameter MoM torques, except for in a few certain circumstances [165].

### 4.3. Ceramic-on-Polyethylene (CoP)

Ceramic manufacturing has significantly improved in the last 20 years, while the introduction of cross-linked polyethylene was associated with significantly better biomechanical and clinical results. The introduction of aluminium femoral heads reduced rates of linear and volumetric wear in vivo. Kusaba [166] studied frictional torque in 67 prosthetic cups, 30 combined with aluminium heads and 37 with metal heads, and reported that the worn alumina heads remain smoother than the new cobalt chrome heads (0.13 mm/year vs. 0.19 mm/year).

In vivo studies that have evaluated the wear of ceramic on polyethylene fall into two broad groups—those that evaluate only the ceramic–polyethylene couple and those that compare with other biomaterial couples. Urban [167] conducted a 21-year study and highlighted an 80% survival of prostheses at 20 years, with a linear wear rate of approximately 0.034 mm/year. Wroblewski et al. [168] reported mean values of 0.03 mm linear wear at 15 years; Sugano et al. [169] demonstrated 0.1 mm wear/year for the first-generation ceramics.

Studies comparing ceramic-polyethylene versus metal-polyethylene coupling results have produced mixed results. Two larger studies [170,171] highlighted similar wear rates in 200 implanted hips at 5 and 6 years, respectively. Similarly, Cohn demonstrated that there was no difference in wear between zirconium and cobalt-chromium heads at four years [172]. At the same time, Ihle et al. [173] demonstrated a halving of wear in the case of alumina compared to cobalt-chromium at 20 years.

Ceramic-ceramic has lower wear rates when compared to ceramic-polyethylene. However, medium-term studies using alumina-type ceramics with cross-linked polyethylene reported similar osteolysis or patient satisfaction at 5 years [174].

### 4.4. Ceramic-on-Ceramic (CoC)

The ceramic-ceramic tribological couple (ceramic head in a ceramic acetabular liner) combines the properties of a high scratch resistance material with very low friction coefficients, thus reducing the risk of wear, osteolysis, and loosening (Figure 2C,F). Hernigou [175] investigated wear and osteolysis in 28 bilateral hip arthroplasties with 20-year survival; one hip with CoC and the contralateral hip with CoP. The results demonstrated that both the wear surface and the volume of osteolysis were lower in the case of hips with ceramic-on-ceramic-bearing surfaces.

Although the release of wear particles is the smallest of all tribological couples, the ceramic-ceramic couple also has some disadvantages and risks: squeaking and ceramic fracture. Hernigou et al. [176] continued the previous research and, in 2016, described a squeaking rate of 0–33% without finding an exact cause of this noise. It appears that the movement of the ceramic head over a worn zone triggers an inaudible ceramic vibration that can be amplified by the mechanical components of the prosthetic implant, turning it into noise. Other studies incriminate the disruption of fluid lubrication [177], the mismatch between a zirconium head and an alumina liner causing a squeak [178], or the use of a large diameter head in a young patient [179].

If squeaking does not have a major functional impact on the survival of the implant over time, the fracture of the head or the ceramic liner is a major complication that requires prosthetic revision. Among the causes of ceramic fracture are the imperfections of the ceramic material, the malpositioning of the insert in the metal cup and of the cup in the acetabulum [180]. With the development of new generations of ceramics, this risk has decreased significantly [181], with these biomaterials not breaking at a force of 12 kN [182], a force above normal activity. In the case of correctly positioned implants, only a major trauma can cause a fracture of the head or the liner [183].

Malpositioning of the ceramic insert causes difficult intraoperative reduction and excessive wear with risk of fracture of the ceramic insert without trauma [184]. The vertical malposition of the metal cup increases the risk of fracture [185].

The fracture of the ceramic implant must be recognized and operated on early due to the significant metallosis occurring at the contact between the metal neck and the acetabular metalback [176].

## 5. Brief Requirements for THA’s Materials

In brief, the success of THA depends on the orthopaedic surgeon, who not only performs the operation but also chooses the best substitute so that the patient can function properly longer without it being rejected. The choosing process can be affected by the patient’s age, health condition, weight, how the materials behave, and how active the patient is after surgery. As a result, for THA to be successful, the biomaterial employed must be biomechanically compatible and have a modulus comparable to the bone, ranging from 4 to 30 GPa [186]. Consequently, to minimize implant loosening and reduce the need for recurrent surgery, an optimal combination of high strength and low modulus closer to that of bone must be employed for the material implant [186]. Furthermore, the implant materials should be extremely nontoxic, with no allergic or inflammatory responses in human tissue and cells, as stated by the material’s biocompatibility [187]. The biocompatibility of a material is influenced by the host reaction, induced through the implant itself and materials resulting from degradation. A material used in THA’s cytotoxicity is often tested in vitro on bone-specific cell lines and fibroblasts using a cell viability assay, and if the cytotoxicity is less than 75–70%, the material does not qualify as a biomaterial used in THA. On the contrary, when cytotoxicity approaches 100%, the material becomes more biocompatible [20,41,68,98,186,187,188,189,190]. In conclusion, following hip arthroplasty, the prosthesis may need to be replaced for the following main reasons: (a) wear such as severely damaged or aseptically loosening the implant surrounding tissues, (b) mechanical damage such as fracturing or entirely worn of the implant, (c) appearance of strongly localized infection surrounding the implant, or (d) other causes such as implant instability or dislocation [189].

## 6. Conclusions

Total hip arthroplasty, although very successful, is not without possible complications. Survival over time depends on factors related to the patient and the surgeon’s experience and surgical technique, but not least on the quality and combination of biomaterials used. The characteristics of the different bearing surfaces and the controversial studies in the literature make it difficult to choose the best implant. The CoC bearing surface shows the least wear, but at a high cost, with the risk of fracture and squeaking. CoP has a higher wear rate than CoC; however, it is cheaper but more expensive when compared to MoP. MoP has the lowest price but the highest wear rate, higher than that of MoM. MoM has a high cost, and the risk of pseudotumors and high levels of metal ions in serum and urine means it is no longer recommended.

There is no such thing as a perfect implant, but there is an ideal implant for each patient. Implant-to-patient matching and implant selection is a difficult task that should consider multiple factors like bone quality and general health status. A good initial choice of implant will decide its survival rate and, consequently, the patient’s satisfaction, and will minimize both social and economic costs generated by the subsequent revision of the total hip prosthesis.

The development of new biomaterials combining the use of new nanoparticles with antimicrobial activity to reduce the risk of infection, among other improvements, will increase the success of these complex surgical interventions and the long-term survival of the implant.

## Figures and Tables

**Table 1:** Advantages and disadvantages of polymers in THA.

Polymer	Advantages	Disadvantages	References
Polymethyl methacrylate	Long-term studies showed >90% survival at 10 years	The accelerator and the monomer are suspected to cause the loosening of the implant Stick the mobile components of the prosthesis	[48,49,50,51,52]
Polytetrafluoroethylene [PTFE]	- thermally stable	- very high wear rates	[20,32,53,54,55]
	- hydrophobic		
Ultra-high molecular weight polyethylene [UHMWPE]	- good wear resistance	- the release of polyethylene wear particles	[17,18,32,54,56,57,58,59,60,61,62,63,64,65,66]
	- high strength		
	- biocompatibility		
Highly cross-linked polyethylene [HXLPE]	- less release of free radicals	- compromising the mechanical properties of UHMWPE: hardness and rigidity	[20,36,37,45,54,67,68,69,70,71,72,73,74,75,76,77,78,79,80,81]
	- higher wear resistance		
Vitamin E-blended polymers	- higher wear resistance than UHMWPE	- insufficient clinical studies	[38,39,40,54,59,82,83,84,85,86,87,88,89]
Polyether-ether-ketone [PEEK]	- decreases the production of wear particles	- insufficient clinical studies	[90,91,92]
	- decreasing the stress shielding effect (in stems)		
Poly 2-methacryloyloxyethyl phosphorylcholine [PMPC]	- decreases the production of wear particles and bone resorption responses	- insufficient clinical studies	[54,93]
Polycarbonate-urethane [PCU]	- biostability	- insufficient clinical studies	[28,54,94]
	- hydrolysis resistance, oxidation, and calcification		
	- absent biodegradation		
	- low wear rate		
	- high resistance to corrosion		

**Table 2:** Advantages and disadvantages of metals in THA.

Metals	Advantages	Disadvantages	References
Stainless steel	- resistance to oxidation	- poor biocompatibility	[11,45,46,54,95,98]
	- easy fabrication	- abrasive wear	
Cobalt-chromium molybdenum [CoCrMo] alloys	- high corrosion resistance	- the particles released in the joint cause an inflammatory reaction and subsequently osteolysis	[54,83,116]
Titanium alloys [Ti-6Al-4 V]	- biocompatibility	- not for the femoral head - low wear resistance	[54,98,117,118,119]
	- resistance to corrosion		
	- high values of mechanical strength		
Zirconium alloy [Zr-2.5Nb]	- increased hardness and low roughness similar to ceramics	- insufficient clinical studies	[99,100,101,102,103,104,105,120]
	- increased resistance to fatigue and breakage similar to metal		
	- the decrease in the release of particles in the joint compared to CoCrMo		
Polycrystalline Diamond	- excellent hardness, extreme wear resistance, low coefficient of friction, superior toughness, and good biocompatibility, good dimensional stability and resistance to deformation and mechanical damage	- insufficient clinical studies	[111,121,122,123,124,125,126]
Magnesium alloy (AZ31-Si3N4 Alloy)	- excellent biocompatibility	- insufficient clinical studies	[112]

**Table 3:** Advantages and disadvantages of ceramics in THA.

Ceramic	Advantages	Disadvantages	References
Alumina	- high wear resistance	- low mechanical strength with risk of breakage	[131,132,133]
	- low coefficient of friction	- squeaking	
Zirconia	- good mechanical proprieties		[54,134]
	- lower wear rate		
	- good resistance to breaking		
Zirconia-toughened alumina [ZTA]	- good strength	- squeaking	[128,131,132,135,136,137]
	- high wear resistance		
Delta ceramic 82% alumina and 17% zirconia	Mid-term large cohort study of revision: - no ceramic fracture	- squeaking rate of 1.7%	[130]
	- survival rate of 91.6% after 12 years		
Sapphire	- high wear resistance	- insufficient clinical studies	[54,138]
	- low coefficient of friction		

## Data Availability

Not applicable.
